# Participation dynamics of a cohort of drug users in a low-threshold methadone treatment programme

**DOI:** 10.1186/s12954-015-0072-z

**Published:** 2015-10-16

**Authors:** Tsz Ho Kwan, Ngai Sze Wong, Shui Shan Lee

**Affiliations:** Jockey Club School of Public Health and Primary Care, The Chinese University of Hong Kong, Shatin, Hong Kong; Stanley Ho Centre for Emerging Infectious Diseases, The Chinese University of Hong Kong, Shatin, Hong Kong

**Keywords:** Low threshold, Methadone maintenance treatment, Injecting drug users, Social connectivity

## Abstract

**Background:**

The low-threshold methadone maintenance treatment (MMT) programme in Hong Kong has been in place for about 40 years. Assessment of the participation pattern of methadone users may inform future programme development to achieve effective harm reduction.

**Methods:**

Longitudinal clinical data of methadone users who first registered for MMT in the year 2008 in Hong Kong were retrieved after ethical and institutional approval. Participation pattern of this cohort was evaluated by examining users’ frequency of attendance and then the overall retention rate. A subgroup of consistent users who remained on treatment in 2012 and/or 2013 was analysed. Comparison was made between high- and low-frequency users, and among high/moderate and low consistency users, to test their correlations with socio-demographics and social connectivity.

**Results:**

The cohort of methadone users registering in the year 2008 was composed of 351 persons, 77 % of whom were ethnic Chinese, with a median age of 34 and the duration of heroin dependency of 6 years. The participation pattern of methadone users was highly variable, with a 6-year retention rate of 38 %. Discontinuations or 'breaks' of >28 days had occurred in 212 (60 %) methadone users. About one third (*n* = 117) were high-frequency users who had attended more than twice a week for at least 90 % of their treatment periods. The dosages received by high-frequency users were generally higher. Of those continuing on treatment in the fifth and/or sixth year (*n* = 185), 30 (16 %), 29 (16 %) and 126 (68 %) gave a high, moderate and low level of consistency as defined by the lengths of breaks. High/moderate consistency users had a longer history of heroin use and a higher degree of connectivity with other users by social network analysis.

**Conclusions:**

Despite the variability of frequency and consistency of attendance of drug users enrolling in the low-threshold MMT programme in Hong Kong, a consistent pattern could be seen in the proportional distribution of dosage and participation efforts. Whereas an adequate dosage was a potential predictor of optimal frequency of attendance, demographics and connectivity had varied between continued users with different levels of consistency.

## Background

Since the early 1990s, harm reduction has been progressively adopted as a strategy to reduce human immunodeficiency virus (HIV) transmission among injecting drug users (IDU) [1]. Through methadone maintenance treatment (MMT) and/or needle exchange programmes (NEP), effectiveness of harm reduction has been proven and thoroughly reviewed in the scientific literature [2]. In Hong Kong, a metropolitan city with a population of over 7 million, MMT has been in place since the mid-1970s, years before HIV/AIDS was discovered as a health threat to IDU [3]. The HIV prevalence in IDU has remained low at <1 %, some 40 years after the programme was set up [4]. As there is no NEP in the territory of Hong Kong, we make an assumption that harm reduction effects on IDU, if proven, are almost exclusively attributable to MMT, a programme currently accessible to some 10,000 attendees per year in the last decades.

The provision of MMT to drug users in Hong Kong is often referred as a low-threshold programme as characterised by its mode of operation [5]. A drug user can present at any of the 20 methadone clinics distributed in different districts in the territory for methadone treatment [6]. These clinics operate 7 days a week and at least 7 hours per day in providing a daily dose of methadone under supervision. MMT is accessible to anyone who drops in without referral, and treatment can be given on the same day of registration. The treatment is delivered at a nominal charge (HK$1 or US$0.13) per day. There is no limitation on the duration of treatment. A person can voluntarily discharge him/herself from the programme and return to it whenever one wants, without any punitive consequence or tedious administrative procedures. Such a low-threshold programme removes barriers to treatment and allows high flexibility for achieving harm reduction in methadone users. Conversely, however, infrequent participation in MMT as allowed by programme flexibility could potentially undermine the harm reduction efforts. An understanding of the participation pattern of IDU registered in a low-threshold MMT programme would be important in evaluating its impacts on the prevention of HIV and blood-borne infections.

Against this background, we undertake to assess the variability of methadone users’ participation efforts in Hong Kong in order to understand the public health impacts of a low-threshold MMT programme. As a territory-wide computerised methadone treatment information system was set up in 2007, useful individual level data spanning over years is now available for our assessment. Complete records of a cohort of drug users who registered over a specified period could be retrieved to study the temporal pattern of participation. In this study, we focused on assessing the associations of participation pattern and one’s socio-demographics and connectivity in the community. The correlation between methadone access patterns and dosage actually received by methadone users was also investigated.

## Methods

Anonymous clinical data from methadone users who registered for the first time in the year 2008 (2008 cohort) were accessed and tracked through the end of 2013. Data fields used in the study included socio-demographics (year of birth, gender, ethnicity, residency status and residential location down to building/district level), drug history, admission history (date of admission and readmission, clinic attended) and daily records of methadone use (date and time of treatment, dispensed dosage, clinic attended). In Hong Kong, local identity cards (HKID) are issued to residents who are permitted to stay for more than 180 days and those who have right of abode. In this study, we defined all HKID holders as local residents, who could be temporary or permanent, but excluding visitors.

In evaluating methadone users’ participation pattern, we examined their frequency of attendance, consistency of participation, and retention rate. High frequency was defined as an attendance of more than twice a week for at least 90 % of their treatment periods. As the MMT programme requires a user to re-register if one discontinues treatment for more than 28 days, this interval (‘break’) was factored in assessing consistency of participation. We defined three levels of participation consistency: high level referring to the absence of any 28-day breaks and therefore not requiring re-registration; moderate level users had at least one break lasting for no more than 3 months, whilst low-level users had breaks lasting over 3 months. Retention refers to the use of methadone for 6 years since programme admission. Association between participation pattern and socio-demographic data, service mode and dosage intake were evaluated by odds ratio (OR) with their corresponding 95 % confidence intervals (CI). Continuous variables were evaluated by Mann-Whitney *U* test. A *p* value <0.05 was considered statistically significant. Analyses were conducted using IBM SPSS Statistics 21.

We evaluated the connectivity of methadone users by conducting spatial and network-based analysis. Spatially, we assessed the geographic relationship between methadone user’s residential location and methadone clinics. There are 18 geographic districts in Hong Kong, so one means of spatial analysis was to determine if a methadone user lived in the same district as that of the clinic attended. Straight line distance was used to measure the distance. Spatial correlation was analysed using ArcGIS 9.3. To explore the networking between methadone users, we assumed that two users were socially connected if they had attended the same clinic within 15 minutes and if such situation happened during at least 10 % of their attendances. In the ensuing social network analysis, weights of linkages between users (edges) were given by an F-score, which we defined as the double of harmonic mean of the fraction of their number of attendances that they met. Edges with F-score lower than 0.1 were excluded. We focused on two metrics: degree and betweenness centrality [7]. Degree is the number of connections between a user and the others. Betweenness centrality is the summation of total number of shortest paths between all pairs of nodes which pass through that node (i.e. user) divided by the total number of corresponding number of possible shortest paths [8]. Social network analysis was conducted using Gephi.

This study was approved by the Joint Chinese University of Hong Kong—New Territories East Cluster Clinical Research Ethics Committee. Institutional approval for data access was obtained from the Department of Health, Hong Kong Special Administrative Region Government, in compliance with the Personal Data (Privacy) Ordinance.

## Results

**Fig. 1:**
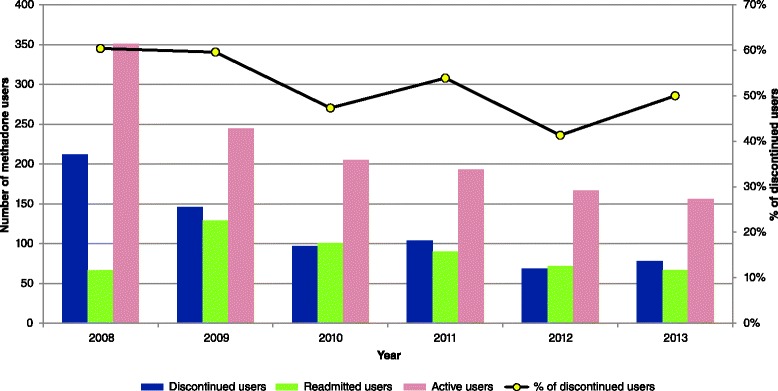
Longitudinal pattern of attendance status of methadone users admitted in 2008 (*n* = 351). Discontinued users had discontinued methadone treatment for >28 days (‘break’), including those who did not return (defaulted). Readmitted users refer to those who returned to the MMT programme after a 28-day break. Active users of the year had attended at least once in the respective year

**Fig. 2:**
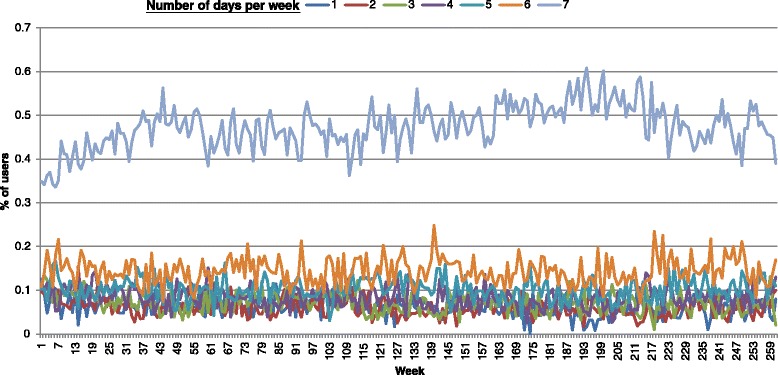
Longitudinal pattern of weekly attendance frequency of methadone users in the cohort. The attendances were classified by the number of days per week, from once daily to once weekly

A total of 11,405 drug users had attended the services of Hong Kong’s methadone clinics in 2008, of whom 351 were newly admitted to the programme and therefore constituted the 2008 cohort on which further analyses were made. A majority of methadone users in the 2008 cohort were male (76 %), local residents (76 %) and ethnic Chinese (77 %) (Table 1). Their median age was 34 (interquartile range (IQR) = 28–40), with a median duration of narcotic use of 8 years (IQR, 2–14). The median number of attendances at methadone clinic(s) between 2008 and 2013 was 311 (IQR, 42–1003). The number of clinics attended by methadone users ranged from 1 to 12 (IQR, 1–3). Some 212 (60 %) methadone users had discontinued temporarily for >28 daysin 2008, with 67 re-registering in the same year. The numbers of readmitted and discontinued users were similar since the second year (Fig. 1). The yearly percentage of users who had breaks or defaulted (not returning any time before the end of 2013) was between 41 and 60 %. Overall, the number of discontinued users decreased over time, and the situation had become relatively stable. The median break interval of a user who subsequently became readmitted was 5 months (IQR, 1–15). None had received non-stop daily treatment throughout the follow-up period. The weekly attendance of drug users in the cohort is shown in Fig. 2. The proportional distribution of users by the number of times of attendance (from once weekly to daily) was quite consistent. Around 40 to 60 % users attended everyday in a week, whilst 10 to 20 % attended 6 days a week. Those who attended the clinic(s) one to five times a week accounted for approximately half of all users.

**Table 1:** General characteristics of methadone treatment users first admitted to the programme in 2008 (*n* = 351)

	No.	%
	*Median*	*IQR*
Socio-demographics at first admission		
Male	266	76 %
HKID holder^a^	267	76 %
Ethnic Chinese	269	77 %
Age (years)	*34*	*28–40*
History of narcotic use (years)	*8*	*2–14*
History of injection (years)	*0*	*0–1*
Methadone clinic attendance pattern		
Proportion attending >2/week during treatment period		
<50 %	65	19 %
50–89 %	164	47 %
≥90 %	122	35 %
Timing of attendance		
Morning	60	17 %
Afternoon	100	28 %
Evening	253	72 %
No. of clinics visited	*2*	*1–3*
Longest break (months) (*n* = 321)	*5*	*1–15*
Methadone treatment received		
Completion of detoxification (*n* = 20)	6	30 %
Mode dose (mg)	*40*	*30–50*
Dosage per day (mg)	*6*	*1–21*
Connectivity with clinic and among users		
Connected in social network^b^	168	48 %
Degree^c^	*0*	*0–2*
Attendance at the nearest clinic (*n* = 134)	94	70 %
Same residing and clinic district (*n* = 322)	191	59 %
Distance (m) from home to frequently visited clinic (*n* = 134)	*609*	*264–1470*
Shortest distance (m) from home to clinic (*n* = 134)	*459*	*237–767*

*IQR* interquartile range

^a^HKID holder refers to a person who is permitted to stay in Hong Kong for more than 180 days with the exclusion of visitors

^b^Users who have at least one connection with any other users with F-score not less than 0.1

^c^The number of connections with other users with F-score not less than 0.1

**Fig. 3:**
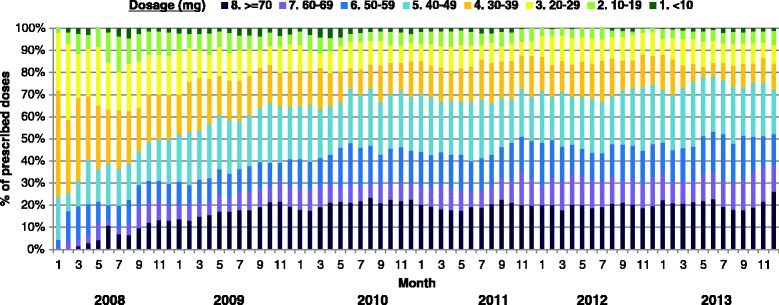
Longitudinal pattern of monthly prescribed doses of methadone users in the cohort. Prescribed dosages were categorised into eight groups: ≥70, 60–69, 50–59, 40–49, 30–39, 20–29, 10–19, and <10 mg

The temporal distribution of daily methadone dosages received by drug users in the 2008 cohort is shown in Fig. 3. Similar to the frequency of attendance, the relative proportion of prescribed dosages dispensed monthly was pretty consistent since the second year. Overall, about 30 % of the dosages were less than 40 mg, whilst some 30 % were ≥60 mg. A small number (20/351 or 5.7 %) of methadone users in the 2008 cohort chose to join the detoxification programme. Of these, six had completed the programme and were not included in the subsequent analyses on dosages and participation patterns. Overall, the median of mode dose of the cohort (*n* = 345) was 40 mg (IQR, 30–50). Some 26 % had ever received a dose of at least 80 mg, whilst 18 % had been on a dose less than 10 mg. A total of 117 (33.3 %) users attended methadone clinics at a high frequency (Table 2) as defined under methodology. They were less likely Chinese (OR = 0.44, *p* = 0.001), local residents (OR = 2.40, *p* = 0.003) and aged over 35 (OR = 1.59, *p* = 0.04). Comparing between the two groups, the mode, median and mean dose was marginally higher in high-frequency users (*U* = 10953, *p* = 0.006; *U* = 11044, *p* = 0.009; *U* = 11117, *p* = 0.01, respectively). Their highest doses were more likely to be ≥80 mg (OR = 2.05, *p* = 0.004) whilst the lowest doses were ≤20 mg (OR = 1.70, *p* = 0.02). For high-frequency users, a dose of >40 mg was consumed in a higher proportion (41 vs. 17 %) of days (*U* = 9996, *p* < 0.001). The dose range among them was higher than those in the low-frequency group (*U* = 9549, *p* < 0.001).

**Table 2:** Comparison of methadone users by frequency of clinic visits (*n* = 345)

	Low frequency (*n* = 228)		High frequency^a^ (*n* = 117)		Test		
	No.	%	No.	%	OR	95 % CI	*p* value
	Median	IQR	Median	IQR	Mann-Whitney *U*		
Socio-demographics at baseline							
Male	169	74 %	94	80 %	1.427	0.83–2.46	0.20
Chinese	186	82 %	77	66 %	0.44	0.26–0.72	0.001*
HKID holder^b^	162	71 %	100	86 %	2.40	1.33–4.32	0.003*
Age (years)	*33*	*28–39*	*35*	*28–41*	*12,066*		0.15
Aged >35	87	38 %	58	50 %	1.59	1.02–2.50	0.04*
Years of narcotic use	9	*3–14*	*7*	*2–15*	*12,643*		0.43
Methadone treatment dosage							
Mode dosage (mg)	*40*	*30–40*	*40*	*30–63*	*10,953*		0.01*
Highest dose (mg)	*50*	*35–75*	*65*	*50–80*	*10,154*		<0.001*
Highest dose ≥80mg	49	22 %	42	36 %	2.05	1.25–3.35	0.004*
Lowest dose (mg)	*20*	*10–30*	*15*	*10–25*	*11,385*		0.02*
Lowest dose <20 mg	91	40 %	62	53 %	1.70	1.08–2.66	0.02*
Dose range (mg)	*35*	*15–52*	*50*	*28–64*	*9549*		<0.001*
Dose range >40 mg	95	42 %	77	66 %	2.70	1.70–4.29	<0.001*
Mean dose (mg)	*37*	*29–48*	*40*	*31–56*	*11,044*		0.01*
Median dose (mg)	*40*	*30–48*	*40*	*30–60*	*11,117*		0.01*
Proportion of days with dose <20 mg	*0 %*	*0–4 %*	*0 %*	*0–7 %*	*11,865*		0.07
Proportion of days with dose >40 mg	*17 %*	*0–65 %*	*41 %*	*7–78 %*	*9996*		<0.001*

OR odds ratio, 95 % CI 95 % confidence interval, *IQR* interquartile range

*p < 0.05

^a^High frequency refers to attendance >2 times/week for >90 % of period of observation

^b^HKID holder refers to a person who is permitted to stay in Hong Kong for more than 180 days excluding visitors

In this study, we defined defaulters as methadone users who discontinued treatment within a year since admission and did not return anytime subsequently as of the end of 2013. Of the 94 defaulters identified, 70 (74.5 %) were male and their median age was 35 years (IQR, 28–41) (Table 3). Defaulters were more likely to be Chinese (OR = 1.92, *p* = 0.04) and less likely HKID holders (OR = 0.24, *p* < 0.001). They also had a longer history of narcotic use (median = 11 years; IQR, 3–15) (*U* = 9878, *p* = 0.02). After further excluding methadone users opting for ‘detoxification’, 185 had attended the methadone clinic(s) at least once in the fifth and/or sixth year of the observation period, qualifying them to be analysed as drug-dependent persons on continued methadone treatment though with different degrees of consistency. Compared to the rest of the cohort, these continued methadone users were more likely to be local residents (OR = 4.35, *p* < 0.001). There was otherwise no significant difference in other socio-demographic characteristics between continued users and discontinued ones who dropped out in or before 2011 (statistics not shown). Among the discontinued users, 72 (21 %), 37 (11 %), 23 (7 %) and 28 (8 %) left the programme in 2008, 2009, 2010 and 2011, respectively. Discontinued methadone users were more likely to have taken a lower mode dose (35 mg vs. 40 mg, *U* = 9789, *p* < 0.001) of methadone. Among the 185 continued methadone users, 30 (16 %) could be classified as achieving a high level of consistency, whilst 29 (16 %) and 126 (68 %) gave a moderate and low level, respectively. Comparisons between high/moderate and low consistency users are shown in Table 4. Methadone users in the high/moderate consistency group were statistically more likely to be aged above 35 at first admission (OR = 3.15, *p* < 0.001) (Table 4a). They gave a longer history of narcotic use (*U* = 2866, *p* = 0.01) and a higher frequency of attendance during treatment. High/moderate consistency users were less likely to attend methadone clinics in the evening (OR = 0.32, *p* = 0.001) but more likely to attend in the morning (OR = 3.60, *p* < 0.001) (Table 4b). They were also less likely to have visited more methadone clinics (*U* = 3065, *p* = 0.05). The average doses (mean, mode and median) of methadone, the dose range and highest/lowest doses did not differ between the two groups significantly (Table 4c).

**Table 3:** Comparison between drug users who continued to receive methadone for more than 1 year and defaulters who discontinued treatment within 1 year (*n* = 345)

	Defaulters (*n* = 94)		Non-defaulters (*n* = 251)		Test		
	No.	%	No.	%	OR	95 % CI	*p* value
	Median	IQR	Median	IQR	Mann-Whitney *U*		
Baseline socio-demographics							
Male	70	75 %	193	77 %	0.88	0.51–1.52	0.64
Chinese	79	84 %	184	73 %	1.92	1.03–3.56	0.04*
HKID holder^a^	52	55 %	210	84 %	0.24	0.14–0.41	<0.001*
Age (years)	*35*	*28–41*	*34*	*28–39*	*11,105*		0.40
Aged >35	43	46 %	102	41 %	1.23	0.76–1.99	0.73
Years of narcotic use	*11*	*3–15*	*7*	*2–14*	*9878*		0.02*
Connectivity patterns							
Attended the nearest clinic^b^	21	64 %	73	72 %	0.67	0.29–1.54	0.35
Same residing and clinic district^b^	22	67 %	65	64 %	1.11	0.48–2.54	0.81
Distance (m) from home to frequent clinic^b^	*760*	*459–1836*	*498*	*223–1427*	*1311*		0.07
Shortest distance (m) from home to clinic^b^	*520*	*271–881*	*443*	*216–749*	*1450*		0.26
Connected in social network^c^	28	30 %	137	55 %	0.35	0.21–0.59	<0.001*
Degree^d^	*0*	*0–1*	*1*	*0–4*	*8137*		<0.001*
Betweenness centrality^e^	*0*	*0–0*	*0*	*0–42*	*9596*		<0.001*

*OR* odds ratio; *95 % CI* 95 % confidence interval, *IQR* interquartile range

*p < 0.05

^a^HKID holder refers to a person who is permitted to stay in Hong Kong for more than 180 days excluding visitors

^b^Defaulters *n* = 33, non-defaulters *n* = 101

^c^Users who have at least one connection with any other users with F-score not less than 0.1

^d^The number of connections with other users with F-score not less than 0.1

^e^Defaulters *n* = 28, non-defaulters *n* = 137

**Table 4:** Comparison of users who had continued to receive methadone from 2008 to 2012/13 by consistency (*n* = 185)

	Low consistency (*n* = 126)		High/moderate consistency (*n* = 59)		Test		
	No.	%	No.	%	OR	95 % CI	*p* value
	Median	IQR	Median	IQR	Mann-Whitney *U*		
(a) Baseline socio-demographics							
Male	95	75 %	46	78 %	1.16	0.55–2.41	0.70
Chinese	94	75 %	50	85 %	1.89	0.84–4.27	0.12
HKID holder^a^	107	85 %	55	93 %	2.44	0.79–7.53	0.11
Age (years)	*33*	*28–39*	*38*	*31–43*	*2629*		0.001*
Aged >35	46	37 %	38	64 %	3.15	1.65–6.00	<0.001*
Years of narcotic use	*6*	*2–13*	*9*	*3–17*	*2866*		0.01*
(b) Methadone clinic attendance pattern							
No. of attendances	*655*	*295–1081*	*1509*	*1165–1764*	*1037*		<0.001*
Frequency >2/week							
<50 %	13	10 %	2	3 %	0.31	0.07–1.40	0.11
50–89 %	68	54 %	19	32 %	0.41	0.21–0.78	0.01*
≥90 %	45	36 %	38	64 %	3.26	1.71–6.21	<0.001*
Visiting time							
Morning	18	14 %	22	37 %	3.60	1.73–7.38	<0.001*
Afternoon	28	22 %	18	31 %	1.54	0.77–3.08	0.22
Evening	99	79 %	32	54 %	0.32	0.17–0.63	0.001*
No. of clinics visited	*3*	*2–4*	*2*	*2–4*	*3065*		0.05
No. of breaks	*3*	*2–5*	*0*	*0–1*	*0.59*		<0.001*
Longest break (months)	*15*	*8–25*	*1*	*0–2*	/		/
(c) Dosage of methadone							
Highest dose ≥80 mg	47	37 %	19	32 %	0.80	0.42–1.54	0.50
Lowest dose <20 mg	66	52 %	37	63 %	1.53	0.81–2.88	0.19
Dose range (mg)	*51*	*40–61*	*50*	*35–60*	*3332*		0.25
Mode dose (mg)	*40*	*30–60*	*40*	*35–50*	*3603*		0.73
Mean dose (mg)	*43*	*36–55*	*40*	*34–50*	*3402*		0.35
Median dose (mg)	*40*	*35–55*	*40*	*35–50*	*3441*		0.41
Highest dose (mg)	*70*	*55–80*	*65*	*50–80*	*3277*		0.19
Lowest dose (mg)	*15*	*10–25*	*15*	*10–20*	*3439*		0.41
Proportion of days with dose <20 mg	*0 %*	*0–6 %*	*1 %*	*0–7 %*	*3269*		0.17
Proportion of days with dose >40 mg	*48 %*	*17–74 %*	*38 %*	*8-73 %*	*3447*		0.43
(d) Connectivity patterns							
Attended the nearest clinic^b^	37	77 %	15	63 %	0.50	0.17–1.44	0.19
Same residing and clinic district^b^	31	65 %	13	54 %	0.65	0.24–1.76	0.39
Distance (m) from home to frequent clinic^b^	*454*	*188–971*	*584*	*297–2876*	*492*		0.31
Shortest distance (m) from home to clinic^b^	*421*	*182–726*	*481*	*277–778*	*509*		0.42
Connected in social network^c^	70	56 %	40	68 %	1.68	0.88–3.22	0.11
Degree^d^	*1*	*0–4*	*2*	*0–39*	*2888*		0.01*
Betweenness centrality^e^	*1*	*0–7120*	*492*	*0–19906*	*1113*		0.06

*OR* odds ratio, *95 % CI* 95 % confidence interval, *IQR* interquartile range

*p < 0.05

^a^HKID holder refers to a person who is permitted to stay in Hong Kong for more than 180 days excluding visitors

^b^Low consistency *n* = 48, high/moderate consistency *n* = 24

^c^Users who have at least one connection with any other users with F-score not less than 0.1

^d^The number of connections with other users with F-score not less than 0.1

^e^Low consistency *n* = 70, high/moderate consistency *n* = 40

Of the 345 users evaluated (after excluding those who joined and completed the detoxification programme), 131 had continued with methadone treatment for at least 6 years, giving a retention rate of 38 %. Geographically, 191 (59.3 %) out of 322 users with residence addresses resided in the same district as their most frequently visited clinic, a majority of whom (94 out of 134 with home addresses at building level, 70.1 %) had visited the nearest clinic (Table 1). The median distance from home to the most frequently visited clinic and the closest clinic was 609 metres (IQR, 264–1470) and 459 metres (IQR, 237–767), respectively. For continued methadone users (*n* = 185), there was no association between consistency of attendance and the choice of clinic locations (OR = 0.50, *p* = 0.19) (Table 4d). In the social network analyses, there were a total of 185,866 edges derived from the interactions among all methadone users attending the clinics, including the 351 users in 2008 cohort, throughout the observation period, of which the F-scores were not less than 0.1. Comparison was made between high/moderate and low consistency users. By our definition, some 40 (68 %) high/moderate level users were connected with any other users compared to that in 70 (56 %) of low level users. High/moderate level users appeared to give a higher degree of connectedness (*U* = 2888, *p* = 0.01) and a higher betweenness centrality among connected users, with the latter showing marginal statistical significance (*U* = 1113, *p* = 0.06).

## Discussion

Our study of a cohort of drug users in Hong Kong showed an extremely variable participation and dosage pattern of MMT which could be attributed to the characteristic mode of operation of the clinical service. The 6-year retention rate of the programme was 38 %, which is comparable to that derived from another study [9], though the criteria might have varied. Evidently, the low-threshold programme has provided a high flexibility for discharge and readmissions. Whilst methadone users can discharge from the programme without consequences, many voluntarily remained in the programme with different level of consistency in treatment over a 6-year observation period. Though uncommon, a small number of discharged users returned for readmission after a long break of more than 5 years. The retention rate of 38 % could arguably be a minimum as some discharged drug users may return after longer interval. As the study was founded on the analyses of anonymous clinic records, the authors did not have access to the reason of discharge, nor the drug dependency history during the discharged periods. We could not exclude the possibility of users engaging in alternative drug rehabilitation programme with harm reduction impacts. As the duration of treatment is unlimited, patients can, on a lifelong basis, benefit from MMT as long as they are willing to remain in the programme. The opportunity of readmitting to the programme provides drug users with a choice to a normal productive life.

As the intensity of participation in MMT may affect the ultimate harm reduction impacts, we attempted to evaluate the participation pattern of methadone users in our cohort by using two different measures, which are their frequency of attendance and consistency of continued treatment. Despite potential shortcomings of our methodological approach, we were able to confirm that frequency of attendance was associated with dosage received. Not only were high-frequency users receiving a higher regular dosage of methadone but also that a higher proportion of high-frequency users’ maximum daily dosage exceeded 60 mg. In fact, a dosage of >40 mg was prescribed in a higher proportion of their treatment days. Elsewhere, an 80-mg dosage had been used to denote adequacy of treatment [10, 11], though previous studies suggested conflicting results on the effects of methadone dosage [12, 13]. Apparently, adequacy of dosages may rely on other factors which may be genetically or even ethnically determined [14–16]. The implications of the dosages in our cohort would need to be further investigated in pharmacokinetic and pharmacogenetic studies. In our cohort, there was also a wider dose range in high-frequency users, probably reflecting the heterogeneity of not just participation efforts but drug dependency states. As early defaulters were included in the cohort, differentiation into high and low frequency could not effectively infer the situation in continued users, though a higher proportion of high/moderate consistency continued users were frequent attendees by the same definition (64 vs. 36 %, OR = 3.26, *p* < 0.001) (Table 4).

Apparently, frequency of participation only reflected an individual’s day-to-day adherence to MMT and could not be used to infer one’s continued participation in treatment. As each dose of methadone is provided at the clinic and needs to be taken under direct supervision, temporary breaks were unavoidable if one was unable to go to the clinic in the circumstances of, for example, adverse weather, physical ill health, hospitalisation and childcare needs. Take-home doses, a strategy currently unavailable in Hong Kong, may allow extra flexibility and had been found to be associated with better adherence in other countries [17]. In evaluating methadone users’ consistency in continued treatment, dosage did not appear as a potential predictor in our study. Out of the 185 methadone users who have been on MMT for up to 6 years, their dosage patterns (average, maximum and minimum) were similar. The major differences between high/moderate and low consistency continued methadone users were their age and length of heroin use as measured at baseline. Age was likely a confounder as older methadone users tended to have taken heroin for a longer period of time. With a median of 9-years heroin history before registration, high/moderate consistency users had probably experienced repeated failures in rehabilitation and were likely to be more committed to methadone once they experienced benefits of its maintenance. Importantly, the history of narcotic use was also a predictor of defaulting from MMT before completing the first year of treatment. Users’ experience in the first year could be important in shaping drug users’ commitment to consistent use of methadone in the long term [18]. Whereas a low-threshold programme normally appeals to drug users with its hustle-free mode of operation, efforts may need to be intensified in supporting newly admitted drug users in order to achieve a higher level of consistency of MMT.

Demographics aside, we endeavoured to explore the impacts of methadone users’ social connectivity on their consistency of continued treatment. Spatial location of the clinics is one such factor as we have previously shown that methadone users normally attended clinics which were easily accessible by local transport system [6]. Our results showed that low-consistency methadone users had used more clinics, which might have arisen from, or could have led to, their unstable relationship with individual clinical services. Unlike previous studies [19, 20], we could not find any associations between the consistency of continued attendance and distance between home and methadone clinics. This may be attributed to the small size of the city and easy accessibility of most clinics in Hong Kong, a phenomenon which may not hold true elsewhere, especially in rural areas. On the other hand, morning clinics were preferred by older methadone users, which probably reflected the need of younger users to go to work in the daytime. Flexibility of a low-threshold programme therefore allowed methadone users to choose their preferred clinic sessions. Separately, it was reported that social network structure among IDU was associated with HIV infection [21] and HIV risk reduction [22]. In this study, high/moderate consistency users were more likely to be ‘connected’ with more users, and they might serve as hubs in the network. Our observations suggested that peer influence could result in a positive effect in MMT retention. Some IDU gathered in the vicinity of a methadone clinic [23], creating bonding that encouraged them to meet each other, as well as to visit the clinic. As users with higher centrality measures could convey messages more effectively in these gathering places, they could serve important function in supporting the enhancement of harm reduction intensity. We admit however that a cause-and-effect relationship between connectedness and consistency of continued methadone treatment of drug users cannot be confirmed from our results.

Finally, we acknowledge that there are inherent limitations of the study. Firstly, we have relied on the use of anonymous data retrieved from an information system to conduct the analyses. Whereas the frequency of attendance and consistency of continued participation of methadone users were adequately recorded, these were neither linked with their drug dependency states nor the HIV serostatus. Defaulters were specifically excluded in some parts of the analyses as we have no access to the reasons for their departures from MMT programme, nor are we aware of their whereabouts. It is possible that some might have left Hong Kong, become institutionalised (in jail or other drug rehabilitation services) and hospitalised or a small number might have passed away for reasons related or unrelated to drug dependency. Follow-up qualitative studies, as well as data linkage with hospital information system and death registry would fill the data gaps and allow plausible conclusions to be drawn on the relationship between participation effort in MMT programme and health outcomes. Secondly, the observation period was relatively short, understanding that drug dependency could be a lifelong chronic condition. Given a longer period of follow-up observations, some defaulters may return to the programme and would need to be treated as continued users albeit a low level of consistency. Conclusions would be different if this happens. Thirdly, another major limitation was that relationships between users were inferred using a social network methodological approach. Interpretation of the results should be cautioned as we had used repeated concurrent attendance at the same clinic as a surrogate of methadone users’ connectedness. It is possible that ‘linked’ methadone users may not know the other users in person in real life. Nevertheless, the dataset used in the study was a relatively complete one for describing and analysing the methadone usage pattern in a low-threshold MMT programme in the territory of Hong Kong.

## Conclusions

The low-threshold MMT programme in Hong Kong allows drug-dependent users to access methadone with a remarkable level of flexibility which could contribute to facilitating their return to a healthy and productive life. With a 6-year retention rate of 38 %, their participation was characterised by a wide-ranging frequency of attendance and variable consistency of continued treatment. We have demonstrated that adequate dosage was a potential predictor of high frequency of attendance, whilst demographics varied considerably between users with different levels of consistency in continued treatment. Specifically, experience in the first year in methadone treatment was critical for long-term opioid users, which might affect their consistency in participating continuously in the programme. Likewise, the connectivity of methadone users among themselves could also impact the participation pattern of harm reduction programme, the understanding which may inform interventions to improve treatment outcomes.

## Abbreviations

IDU: injecting drug users
MMT: methadone maintenance treatment
NEP: needle exchange programme
HKID: Hong Kong identity cards
HIV: human immunodeficiency virus
